# Labour in Minor Degrees of Pelvic Contraction

**Published:** 1908-08-22

**Authors:** 


### OBSTETRICS.

### LABOUR IN MINOR DEGREES OF PELVIC CONTRACTION.

It is by no means uncommon in patients with minor degrees of pelvic contraction for three or four children to be sacrificed by embryotomy before a proper examination and estimation of the pelvic diameters leads to appropriate treatment in a future labour. This statement will be corroborated by anyone who has charge of a lying-in hospital or a large gynæcological out-patient department, for these patients sooner or later gravitate to hospitals if they belong to certain classes of the population. The chief reason why so many children are thus sacrificed is the absence of a routine examination of all primiparæ, in order that the condition of the pelvis shall be known before the end of pregnancy. It should not be a difficult matter for a medical man who is engaged to attend a primipara to make one thorough examination during pregnancy. If this became customary it would entail no great hardship on the doctor, and patients would be educated to expect it. At present they do not expect it, and no doubt in many instances resent it. The only way to overcome this feeling is to point out the advantages which must accrue to the patient herself, and to the child in cases where the usual routine is departed from in abnormal pelvis.

There can be no excuse for a patient undergoing more than one difficult labour with destruction of the child. Whenever this has occurred once, treatment should be adopted in the next pregnancy to obviate such a result. Whether the patient is a primipara or not the routine examination should be the same, and the treatment adopted must be based upon this. It does not make much difference at what period of pregnancy the examination is made, provided it is not later than the twenty-eighth week, or more roughly the end of the seventh month (if this is a calendar month the date will be rather later than the twenty-eighth week). The object of the examination is to estimate the length of the true conjugate and the lie of the child. Other diameters of the pelvis may be measured and other general considerations taken into account, but for practical purposes the important thing is the conjugate. In the case of contraction of this diameter we should like to know whether the pelvis is flat or generally contracted, but this is often very difficult to ascertain and the distinction requires special skill. For practical purposes it does not matter much; flat pelves are much more common than those with general contraction. The usual method of estimating the true conjugate is to measure the diagonal conjugate (from the lower part of the symphysis pubis to the promontory of the sacrum) with the fingers, and then to subtract half an inch from the number of inches obtained. It is best to perform this measurement with the patient on the side, and to insert two fingers into the vagina if they are short, because the second finger is longer than the index. Very few people can touch the promontory of the sacrum if the pelvis is normal; not because the fingers are too short, but because the posterior vaginal fornix cannot be raised high enough. Hence it is a fair conclusion that there is no contraction of importance if the promontory cannot be reached. If the contraction of the true conjugate is more than a quarter of an inch, the promontory can generally be reached and an estimate made. Although this is a rough method, it is accurate within an eighth of an inch, and it is astonishing how closely three or four men will independently estimate a true conjugate in a given patient. Of the instruments designed for internal measurements the Skutsch pelvimeter is that which gives the best results, but its use is only additional to the fingers and is not in practice much more accurate.

The measurement of the true conjugate is the key to treatment. Practically in this country we have only two alternatives—induction of labour and Cæsarean section. Symphyseotomy, pubiotomy, and hebotomy have never gained much appreciation with us, and certainly they are not operations to recommend to those in family practice. As a general rule, induction of labour will be chosen as best suited to the majority of cases, for Cæsarean section can only be undertaken with confidence by those who are in the habit of constantly performing abdominal operations, not because it is difficult, but because any man who performs an abdominal section must have surgical technique at his finger-ends.

In the bulk of minor degrees of contraction the conjugate diameter will be over $3\frac{1}{4}$ inches. When induction of labour has been decided upon, we cannot fix any absolute date upon which it should be done, because children vary in size and the head must be taken as the index. Roughly, with $3\frac{1}{2}$ inches true conjugate the thirty-sixth week is about the time at which labour should be induced, but the proper treatment is to see the patient a fortnight before this date and ascertain if the head is in the pelvis or above it. If the latter, and the head can be pressed down into the pelvis, allow the pregnancy to go on a fortnight and then try again. When the head will only enter the brim with difficulty, then is the proper time to induce labour. If the head is unfortunately not presenting, it is recommended to alter the presentation by external manipulation so as to produce a vertex. This cannot always be done, especially if there is but little liquor amnii. In this case one can only induce labour at the time which is indicated by the size of the true conjugate and terminate delivery in the breech position.

*(To be continued.)*

